# Trends in SARS-CoV-2 cycle threshold values in the Czech Republic from April 2020 to April 2022

**DOI:** 10.1038/s41598-023-32953-2

**Published:** 2023-04-15

**Authors:** Dita Musalkova, Lenka Piherova, Ondrej Kwasny, Zuzana Dindova, Lubor Stancik, Hana Hartmannova, Otomar Slama, Petra Peckova, Josef Pargac, Gabriel Minarik, Tomas Zima, Anthony J. Bleyer, Martin Radina, Michal Pohludka, Stanislav Kmoch

**Affiliations:** 1 Research Unit of Rare Diseases, Department of Paediatric and Adolescent Medicine, First Faculty of Medicine, Charles University in Prague and General University Hospital in Prague, Prague, Czech Republic; 2 SPADIA LAB, Ostrava, Czech Republic; 3 Faculty of Safety Engineering, Technical University of Ostrava, Ostrava, Czech Republic; 4 Charles University Innovations Prague, Prague, Czech Republic; 5 Regional Authority of the Central Bohemia Region, Prague, Czech Republic; 6 Medirex Group Academy, Trnava, Slovakia; 7 Institute of Medical Biochemistry and Laboratory Diagnostics, General University Hospital and the First Faculty of Medicine of Charles University, Prague, Czech Republic; 8 Section on Nephrology, Wake Forest School of Medicine, Winston-Salem, NC USA; 9 GeneSpector, Prague, Czech Republic

**Keywords:** Diseases, Respiratory tract diseases, Epidemiology

## Abstract

The inability to predict the evolution of the COVID-19 epidemic hampered abilities to respond to the crisis effectively. The cycle threshold (Ct) from the standard SARS-CoV-2 quantitative reverse transcription-PCR (RT-qPCR) clinical assay is inversely proportional to the amount of SARS-CoV-2 RNA in the sample. We were interested to see if population Ct values could predict future increases in COVID-19 cases as well as subgroups that would be more likely to be affected. This information would have been extremely helpful early in the COVID-19 epidemic. We therefore conducted a retrospective analysis of demographic data and Ct values from 2,076,887 nasopharyngeal swab RT-qPCR tests that were performed at a single diagnostic laboratory in the Czech Republic from April 2020 to April 2022 and from 221,671 tests that were performed as a part of a mandatory school surveillance testing program from March 2021 to March 2022. We found that Ct values could be helpful predictive tools in the real-time management of viral epidemics. First, early measurement of Ct values would have indicated the low viral load in children, equivalent viral load in males and females, and higher viral load in older individuals. Second, rising or falling median Ct values and differences in Ct distribution indicated changes in the transmission in the population. Third, monitoring Ct values and positivity rates would have provided early evidence as to whether prevention measures are effective. Health system authorities should thus consider collecting weekly median Ct values of positively tested samples from major diagnostic laboratories for regional epidemic surveillance.

## Introduction

The SARS-CoV-2 infection and COVID-19 pandemic caused a global crisis affecting all aspects of life for every individual in the global population. Individual countries implemented a wide range of mitigation policies to control the spread of the infection and prevent overload of national health systems. The inability to predict trends in the incidence of SARS-CoV-2 infection and to evaluate effects of mitigation policies hampered abilities to respond to the crisis.

The preferred testing method for SARS-CoV-2 infection is the real-time quantitative reverse transcription-PCR (RT-qPCR) test. The positivity of the test is determined by the cycle threshold (Ct) values. The Ct value is defined as the number of PCR cycles required for the fluorescent signal generated by PCR amplification to exceed the background fluorescence level, and the Ct value is inversely proportional to the amount of target nucleic acid in the sample. Previous studies demonstrated that the Ct value was an accurate predictor of RNA viral loads using cultivable SARS-CoV-2 virus from the nasopharyngeal swabs^1^ and Ct values inversely correlated with transmission risk^2^. Low Ct values indicate a high viral load, an acute phase of infection and potentially high infectivity. High Ct values typically occur in the very early phase of infection and during convalescence. Ct values can thus provide information on the stage of an individual's infection (early, active, convalescent) and may be indicative of disease severity and/or infectivity^3–5^. While Ct values are not useful in assessing disease severity individually^6^, Ct values may be helpful in assessing population infectivity and could be a useful epidemiological early-warning indicator for shifts in transmission^7–10^. Also, considerable controversy exists over the susceptibility to SARS-CoV-2 infection in different age and educational stage groups^11^ and on the effect of school closures on disease spread^12^.

In this study we retrospectively analysed data from 2,076,887 RT-qPCR tests that were performed between April 2020 and April 2022 for diagnostic, epidemiologic and preventive indications in nasopharyngeal swabs from 1,280,248 individuals from geographically diverse regions that employed 98% of the national postal codes in the Czech Republic. Samples were tested at one diagnostic center, SPADIA LAB, in Ostrava. We also analysed the results of 221,671 RT-qPCR tests that were performed on saliva samples from 66,434 individuals as a part of a mandatory surveillance testing program at schools from March 2021 to March 2022. Our objective was to identify how Ct values correlated with epidemiologic trends in the community and possibly with applied mitigation policies, with specific emphasis on individual demographic groups according to age and educational level.

## Methods

The study analysed Ct values obtained from clinically validated (CE-IVD certified) RT-qPCR tests for SARS-CoV-2 infection in the accredited SPADIA LAB (https://www.spadia.cz/), which serves individuals, hospitals, schools, companies, municipalities, and other institutions across the Czech Republic. The methods are described in detail in the Supplementary Methods.

Briefly, the presence of SARS-CoV-2 was determined from nasopharyngeal swabs (viRNATrap, *GeneSpector, Czech Republic*) in the general population. For the surveillance school testing RNA from saliva (Salivette, *Sarstedt, Germany*) was used. Testing was performed with the Reverse Transcriptase quantitative Polymerase Chain Reaction (RT-qPCR) method (gb SARS-CoV-2 Combi, *Generi Biotech, Czech Republic*) on a CFX96 System (*Bio-Rad, Hercules, CA, USA*) with previous automated isolation of nucleic acids on magnetic particles (*GeneSpector, Czech Republic*) using KingFisher Flex Purification System (*Thermo Scientific, Waltham, MA, USA*). Data acquired with each test included the date when the sample was obtained, a personal ID, age and gender, and the postal code of the patient’s permanent address. The test was considered positive if the fluorescent signal exceeded the threshold before 38 cycles of the PCR amplification. Information on comorbid conditions and COVID-19 symptoms was not available. Data cleaning, summary statistics and visualization of the dataset was performed in R-studio (R version 4.0.5). Data from surveillance school testing performed in the saliva samples is shown separately, in the Fig. 4 only.

All research was conducted in accordance with the Declaration of Helsinki and all relevant institutional guidelines and ethical regulations for work with human participants.

The Ethics Committee of the Medirex a.s. (approval No. 20187/2022) and the Ethics Committee of the Institute for Clinical and Experimental Medicine and of the Thomayer University Hospital (approval No. 29162/21; G-21-69) approved the study and waived the need to obtain informed consent as the nature of the study was retrospective.

The data were categorized into nine demographic groups according to age and educational level: newborns and toddlers (0–2 years); preschool children (3–5 years); elementary school children I. (6–8 years); elementary school children II. (9–13 years); elementary school children III. (14–15 years); youth I/secondary schools (16–19 years); youth II/universities (20–26 years); adults (27–65 years) and seniors (66 years and over).

Differences of Ct values between categories were compared using the Kruskal–Wallis One-Way Analysis of Variance with a Dunn´s post hoc test. The odds ratios (ORs) and 95% confidence intervals (CIs) for SARS-CoV-2 infection prevalence between categories were determined and compared between categories weekly using the Wald test and Fisher´s exact test. Difference in aggregate prevalence of SARS-CoV-2 positive tests between categories was compared using the median-unbiased estimation (mid-p method) and Fisher´s exact test.

## Results

### Ct values were obtained from ~ 10% of all SARS-CoV-2 tests and regions represented by 98% of all postal codes in the Czech Republic

SPADIA LAB performed approximately 10% of all SARS-CoV-2 testing in the Czech Republic from April 2020 to April 2022, on samples received from 2616 of 2676 (98%) of all of Czech postal codes. During this period the laboratory performed 2,298,558 tests. The population source for the study, the demographic structure of the study population with number of test performed in individual age and educational groups are shown in Supplementary Fig. 1A and 1B, respectively. From April 2020 to April 2022 the laboratory performed 2,076,887 tests for diagnostic, epidemiologic and preventive purposes in nasopharyngeal swabs from 1,280,248 individuals. Of these, 406,786 (20%) tests and 389,656 (30%) individuals were positive for SARS-CoV-2 infection. The number of performed tests fluctuated over the course of the epidemic and peaked during the second and third waves, when there were more than 80,000 tests per week (Supplementary Fig. S2A). 66.9% of individuals were tested once, with 32.7% undergoing multiple testing (2–8 times). 5997 (0.5%) individuals were tested very frequently (9–87 times) (Supplementary Fig. S2B). The number of positive diagnostic tests also fluctuated over the course of the epidemic and peaked during the second and third waves, when it exceeded 15,000 and 30,000 positive tests per week (Supplementary Fig. S2C). 95.9% of individuals who were tested positive had only one positive test result, 3.8% had two positive results, and 0.3% had between 3 and 7 positive results (Supplementary Fig. S2D). The dataset was not trimmed to remove multiple positive tests per individual. The positivity rate also fluctuated over the course of the epidemic and peaked at 45% (Supplementary Fig. S2E).

### Weekly median Ct values estimates the epidemic trajectory and the median Ct values were steadily increasing in the population

**Figure 1:**
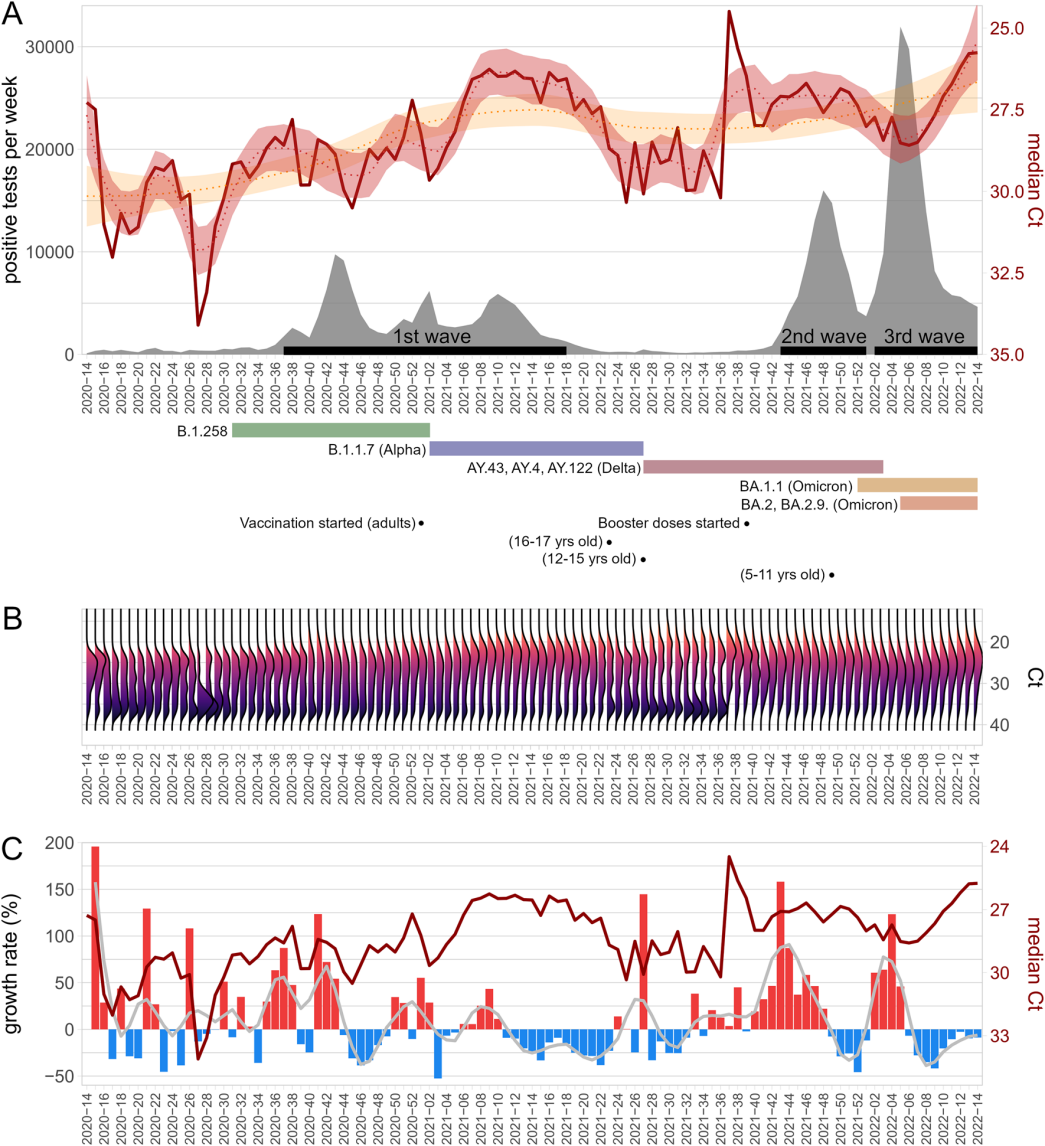
(**A**) The time course of median Ct values per week (dark red line) and the total number of positive tests per week (grey area). Loess curves fitted to Ct medians are shown as a dotted red line (alpha = 0.15) and yellow line (alpha = 0.7). Shaded areas surrounding the curves are 0.95 confidence intervals. Black segments on the x-axis indicate major epidemics waves considered in downstream analyses. The coloured segments below the x-axis indicate the main SARS-CoV-2 variants present in the population based on publicly available data. The black dots indicate the start of vaccination. (**B**) Ct distributions of positive tests shown as half violin plots. (**C**) The time course of median Ct values per week (dark red line) and the growth rate of positive samples. The growth rate is expressed in blue for negative values (decline) and red for positive values (growth). Loess curve fitted to the growth rate is shown as a solid grey line (alpha = 0.1).

The median weekly Ct values (Fig. 1A, B) fluctuated between 24.5 and 34.1 over the course of the epidemic. Lower median Ct values (corresponding to increase in population SARS-CoV-2 load) were associated with a positive growth rate of positive samples and a steep rise in positive cases approximately 3 weeks thereafter, whereas higher median Ct values (corresponding to decrease in population SARS-CoV-2 load) indicated the beginning of a resolution of a recent outbreak (Fig. 1C, Supplementary Fig. S3). This trend disappeared at the end of 2021 because the increase of positively tested samples in January 2022 was on the contrary preceded by an increase of median Ct values. Individual Ct values of positive tests ranged from 12 to 38 cycles and demonstrated a bimodal distribution (Fig. 1B). The bimodality was evident over the course of the epidemic, but the distribution of Ct values changed over time. The proportion of samples with low Ct increased with the proportion of positive tests and decreased when infection waned. Interestingly, a sudden increase in the proportion of samples with low Ct values was associated with an increase in incidence of SARS-CoV-2 infection a few weeks thereafter, as can be seen with the shift between weeks 2021-36 and 2021-38 and start of the 2nd wave four weeks later.

Overall, the median Ct values steadily decreased from approximately 30 to 26.5 over the course of the epidemic. The difference in median weekly Ct values corresponds to approximately ten-fold increase in viral RNA load in analysed materials from the beginning of the epidemic until the present; see Loess curves in Fig. 1A.

### SARS-CoV-2 testing disproportionately aimed at children

**Figure 2:**
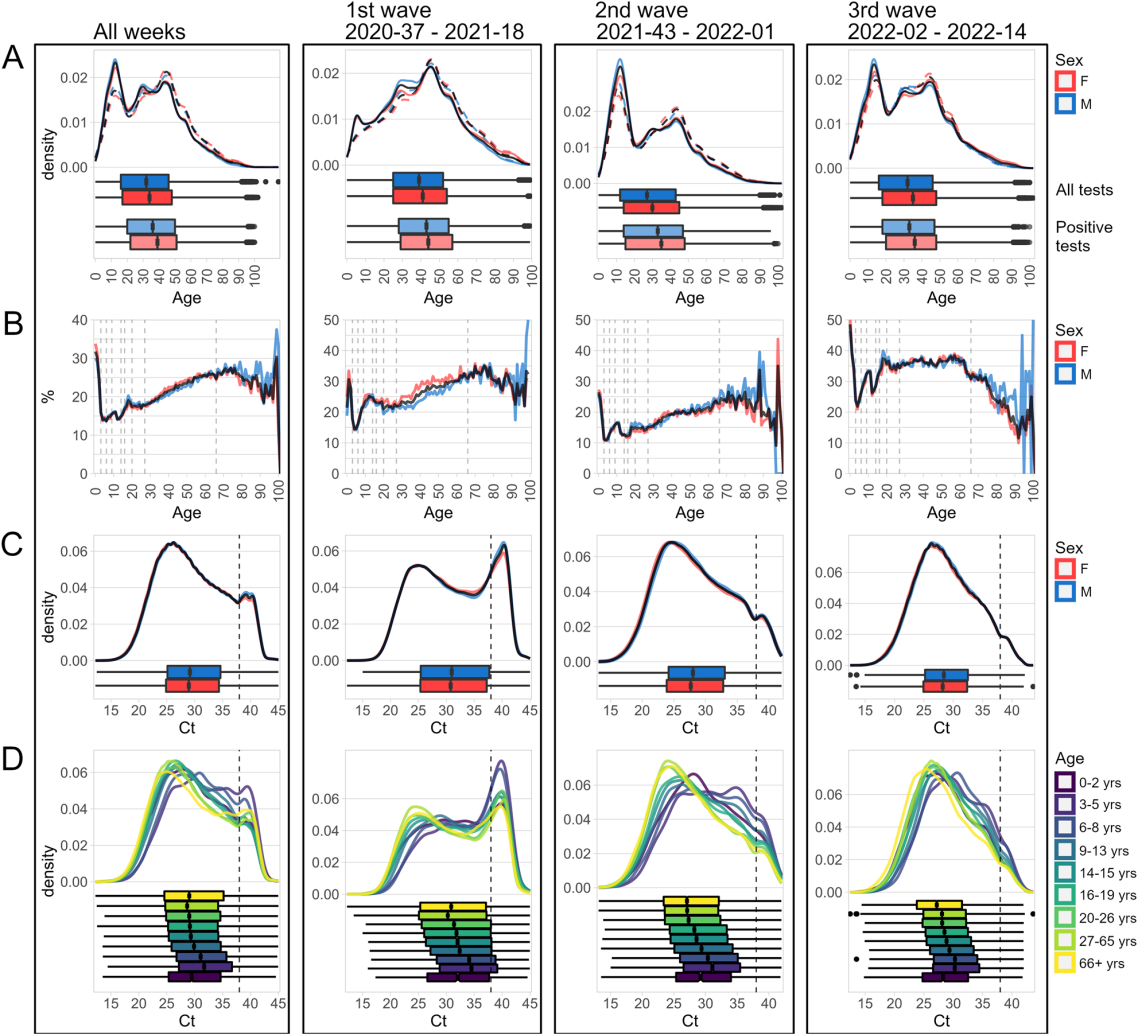
(**A**) Age distributions of all tested individuals; all tests (solid lines and darker boxplots) and positive tests (dashed lines and lighter boxplots). (**B**) Positivity of the tests (in %) in relation to the age of the tested individuals. Dotted vertical lines show limits for the age categories. (**C**) Ct distributions of all tests. Dotted vertical line shows threshold used for the test positivity. (**D**) Ct distributions of all tests. Data is divided according to the age of the individuals. Data in (**A**–**C**) represents all tests (black), tests performed in females (red), and tests performed in males (blue). Data in (**A**, **C**, **D**) is shown as density plots supplemented with boxplots.

Overall and wave-specific age and sex distributions of all tests, positive tests and corresponding test positivity rates are shown in Fig. 2A and B. Compared to the age structure of the population (https://www.czso.cz/staticke/animgraf/cz/), the distribution of all performed tests revealed disproportionally increased testing with lower number of positive tests and lower test positivity rates in children compare to other age groups. This disproportion was not evident in the first wave of the epidemic, when the distribution of all tests, positive tests and corresponding test positivity rates correlated with population structure. It became evident during the second and third waves of the epidemic and was due to surveillance testing of school children. While testing rates were disproportionately high in children, the test positivity rates was constantly lowest in children during the first two waves of epidemics. The trend in positivity rates changed during third wave in 2022 with the Omicron variant, when the lowest test positivity rates were present in the senior population. There were no substantial differences in distributions of all tests, positive tests and corresponding test positivity rates between females and males in any of the age categories.

Overall and wave-specific distributions of Ct values are shown in Fig. 2C**.** During the first wave of epidemics, the Ct values demonstrated a bimodal distribution with peaks at 25 and 40 cycles and a local minimum in between at 35 cycles. The peak around higher Ct values that was evident during the first wave of epidemics gradually disappeared. Distribution of Ct values according to age is shown in Fig. 2D**.** It demonstrates an age dependent decrease in Ct values (eg. higher amount of SARS-CoV-2 RNA load) from children to adults. This pattern remained consistent throughout the epidemic.

### Children and youth had consistently lower SARS-CoV-2 viral load then adults

**Figure 3:**
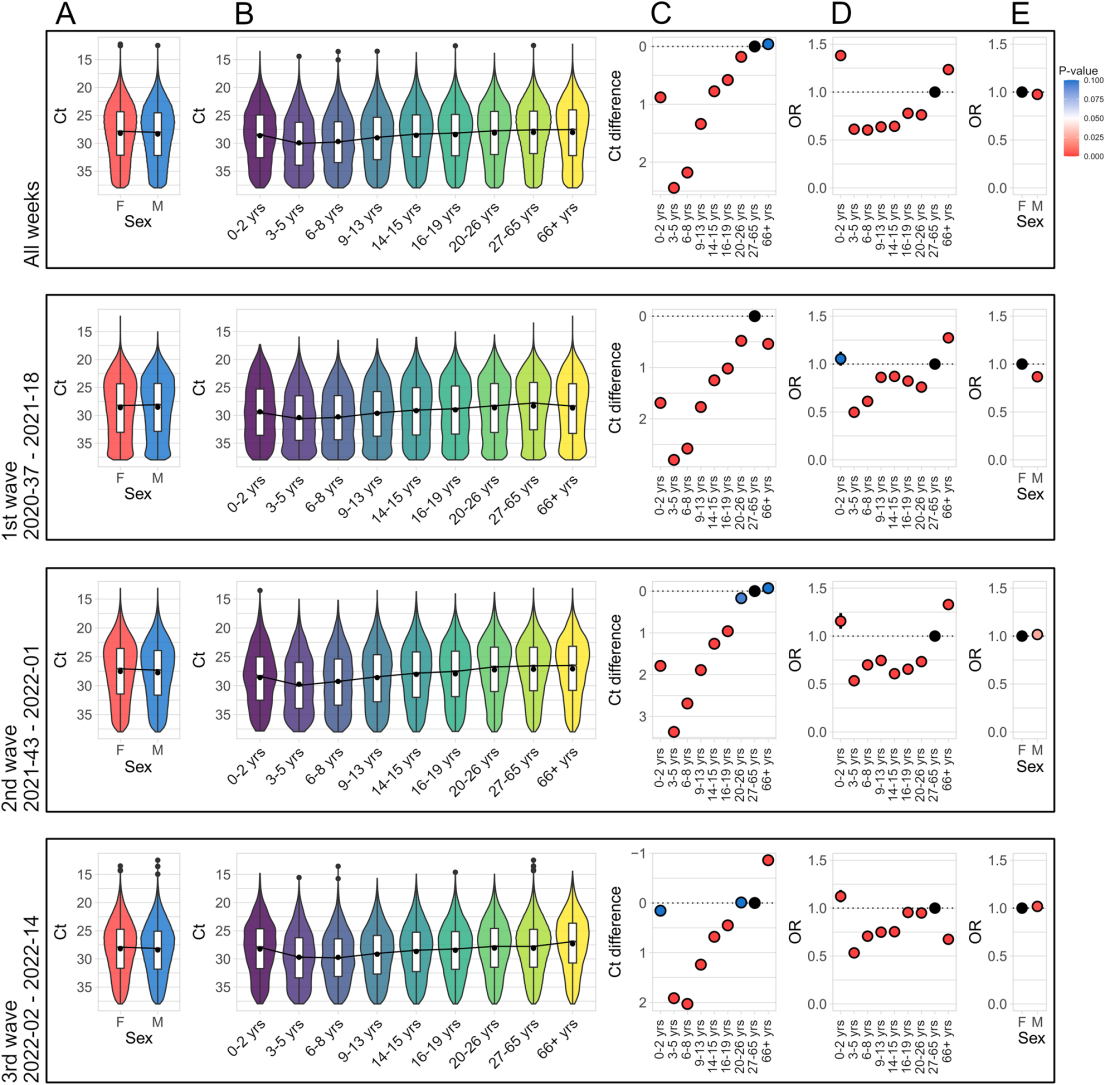
Ct distributions of positive tests categorized according to the sex (**A**) or age of the individual (**B**). Data are shown as violin plots, supplemented with Ct means (black points) and notched boxplots. Medians are connected with a black line. (**C**) Differences of median Ct values (compared to the group of adults 27–65 years). Color of the points corresponds to P-values from Dunn´s test. (**D**) Odds ratios of test positivity rate per age group compared to the group of adults (27–65 years). (**E**) Odds ratios of test positivity rate according to the sex. Confidence intervals were computed using the median-unbiased estimation (mid-p method), color of the points corresponds to P-values from Fisher´s exact test.

Overall and wave-specific differences in mean or median Ct values of positive tests between females and males (Fig. 3A) were minimal (average Ct = 28.22 vs 28.35 and median Ct = 27.87 vs. 28.03, respectively; P-value = 3 × 10^–16^ by Mann–Whitney U test).

Overall and wave-specific distribution of Ct values were significantly different in individual age groups (P-value < 2 × 10^−16^; one-Way Kruskal–Wallis ANOVA with Dunn´s post hoc test). Figure 3B, Supplementary Fig. S4 and Supplementary Table 1 demonstrate that the Ct values were highest (eg. SARS-CoV-2 RNA load was lowest) in children, and that the Ct decreased (eg. SARS-CoV-2 RNA load increased) with age (Fig. 3C). The difference in Ct values corresponds to a 4–5-fold reduction in viral RNA load in analysed materials between children and adults. Supplementary Fig. S5 shows that the weekly median Ct values in individual age groups fluctuated over the course of the epidemic, but the differences in Ct values compared to the group of adults (27–65 years old) were relatively consistent (Supplementary Fig. S6).

### Children had a consistently lower prevalence of SARS-CoV-2 positive tests

We found that children and youths had consistently approximately two-times lower aggregate prevalence of positive tests than adults and seniors (Fig. 3D; Suppl. Table 2). The proportion of positivity rates was also lower in males with the odds ratio of 0.976 (95% CI from 0.969 to 0.982) (Fig. 3E).

Supplementary Fig. S7 shows that the odds ratios of diagnostic test positivity rate per week fluctuated over the course of the epidemic, but the differences compared to the group of adults (27–65 years old) were relatively consistent. The lowest odds ratios of positivity rates (approximately 2-times lower), were in pre-school (3–5 years old) and elementary school I. (6–8 years) children. Seniors (66 years and over) had the odds ratios of diagnostic test positivity rate constantly highest up to the beginning of 2022. Supplementary Fig. S8 shows that school closures were followed by decreased Ct values and an increased weekly odds ratios of diagnostic test positivity rates in populations of elementary school I. (6–8 years) children.

### SARS-CoV-2 infection rates in elementary school children were low even in the setting of high population incidence

**Figure 4:**
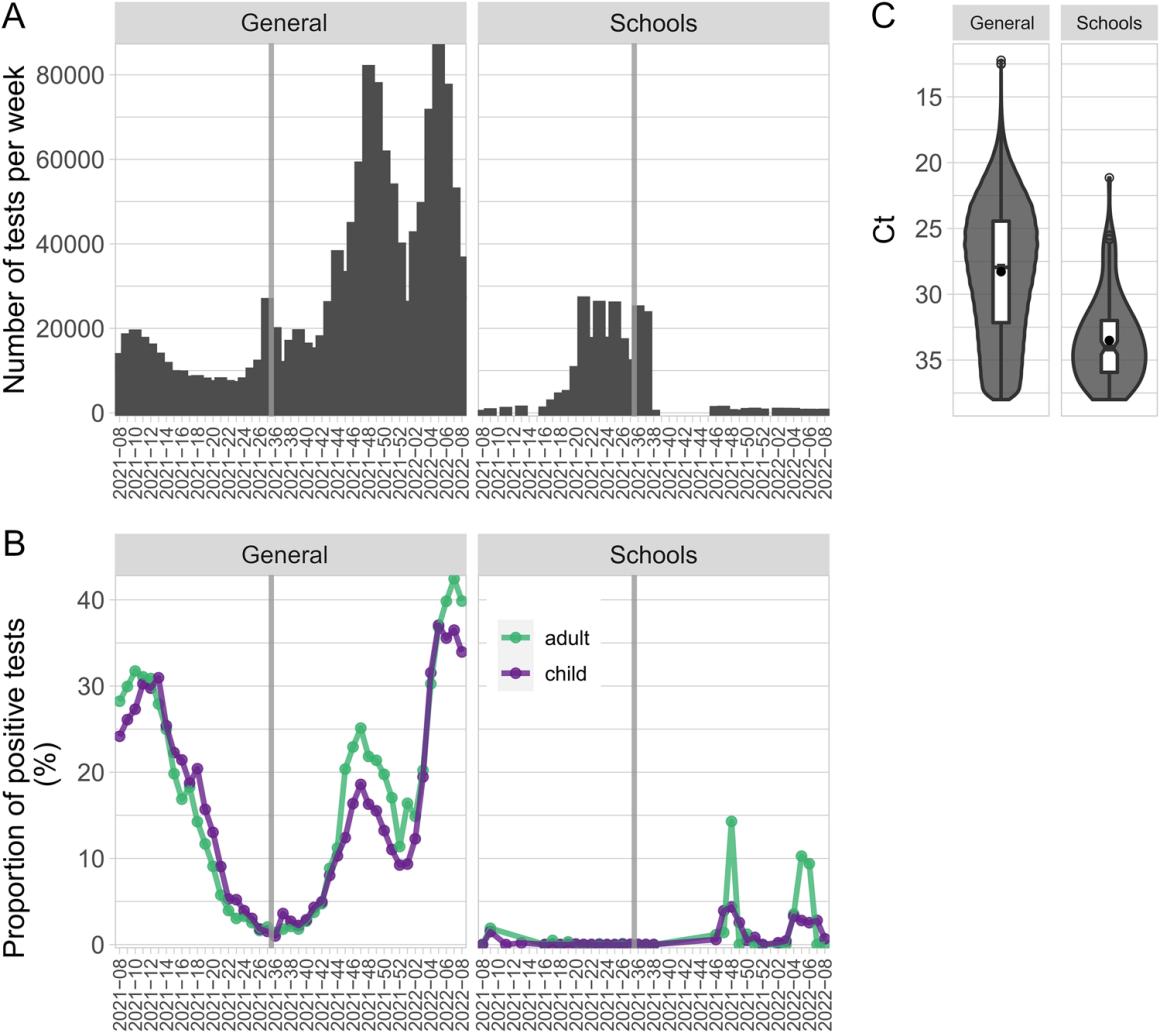
Comparison of general and school testing. (**A**) The total number of general and school tests that were performed between weeks 2021-08 and 2022-08. (**B**) Proportion of positive general and school tests (%). The age of 19 years was used as the threshold between the group of children and adults. (**C**) Ct distributions of positive general and school tests shown as violin plots, supplemented with mean Ct values (black points) and notched boxplots.

To estimate the incidence and prevalence of SARS-CoV-2 infection in elementary schools, the laboratory performed 221,671 saliva tests in 66,434 individuals (63,283 children and 3151 adults) between 2021-08 and 2022-08 as part of a mandatory surveillance testing program at schools. Figure 4A shows time distribution of general testing in the population and school testing. In schools, 156 (0.0007%) tests and 154 (0.002%) individuals were positive for SARS-CoV-2, with 27 of 154 (18%) of positively tested individuals being adults; (OR = 4.3 (95% CI from 2.8 to 6.5; P < 0.0001). In the same period, the laboratory performed 1,307,283 tests in the general population, of which 270,917 (21%) were positive. Figure 4B shows that test positivity rates in the general population across the time window initially decreased from 30 to 2% as the first epidemic wave waned and then increased up to 35% at the beginning of 2022 with the second and third waves. During this time interval, the proportion of positive tests in school children ranged from 0 to 4% (Fig. 4B). In positive school samples the Ct values ranged from 21.15 to 38 cycles (mean Ct = 33.51, median Ct = 34.08); the Ct value distribution was different from diagnostic tests partly because the surveillance tests were performed in saliva (Fig. 4C).

## Discussion

During this epidemic, prediction of surges or declines in COVID-19 cases has not been possible^13^, significantly affecting the ability of public health officials to respond to this crisis. For example, in some areas cancellation of elective surgeries was performed early in the epidemic in anticipation of a rise in cases that did not occur. Similarly, some centers did not cancel elective surgeries and then were overwhelmed by an unexpected increase in COVID-19 cases. In this analysis we sought to investigate how mean weekly Ct values from RT-qPCR tests correlate with the incidence SARS-CoV-2 infection in the general population and individual age groups of the Czech Republic, as the role of age in disease transmission is of great importance in designing the interventions^14,15^.

The Ct values are impacted by many pre-analytical and analytical variables that hamper inter-laboratory comparisons and prevent patient management based on results^6^. Here we compared Ct values in a consistent manner, with samples obtained from nasopharyngeal swabs that were collected and transported in one type of preservation media, using one type of RT-qPCR assay, and performed in a single laboratory that provided on average 10% of all tests across > 98% of postal codes of the Czech Republic. This ensured the highest standardization of the Ct estimation possible. Our study has several limitations, such as unavailable information on the presence and spectrum of COVID-19 symptoms and any comorbidities. Also, we were not able to divide the data according to the testing purposes and SARS-CoV-2 variants. Yet, the Ct values obtained from more than two million samples over the period of two years from a homogeneous population experiencing waves of the Alpha, Delta and Omicron variants across individual demographic groups according to age and educational level provided a unique opportunity for analysis, with statistical power for individual correlations.

First, we analysed the Ct values aggregately with an aim to identify how Ct values correlated with the incidence and course of the epidemic. The proportion of positively tested individuals demonstrated a gender independent increase correlated with the age. The Ct values demonstrated a bimodal distribution that is typical for other viral infections^16^, as well as for SARS-CoV-2^17,18^, where the samples with low Ct values is thought to indicate the proportion of individuals with high viral loads in the acute phase of infection and of potentially high infectivity, while higher Ct values are typical for an early phase of the infection or convalescence^3,19,20^. Aggregate analysis and distribution of Ct values revealed that the mean weekly Ct values fluctuated over the course of the epidemic and that a gradual decrease in the mean weekly Ct values (Fig. 1A and C) and increased dominance of the peak around lower Ct values (Fig. 1B) were indicative of a future increase in the incidence of positively tested samples and individuals in the 1st and 2nd wave. These results were not influenced by the cut-off value for test positivity (Supplementary Fig. S9). This supports recent virologic surveys demonstrating that community/group SARS-CoV-2 Ct values are a useful epidemiological early-warning indicator for shifts in transmission^7–9^. Hay et al.^7^ found that population-level Ct distributions strongly correlate with the estimates for the effective reproductive number or growth rate in real-world settings. However, they were concerned that changes in test availability, testing methods, strategies, and changing viral properties may also lead to shifts in Ct value distributions. In our setup, testing capacities were sufficient and the methods were highly standardized. On the other hand, government measures and restrictions and SARS-CoV-2 variants changed frequently throughout the two years. Also, in December 2020 the vaccination started, but the differences between the Ct values of unvaccinated and fully vaccinated people were negligible (Supplementary Fig. S10). Interestingly, we observed an approximately ten-fold increase in viral RNA load in analysed materials from the beginning of the epidemic until the present. This is suggestive of enhanced transmissibility and partial immune escape of SARS-CoV-2 virus over time^21^. Variants of concern (Alpha, Beta, Gamma, Delta, Omicron) have shown differences in evasion from immunity, viral loads, and incubation and shedding period. Studies reported higher RNA viral load in Alpha variant compared with the ancestral virus and even higher increase of RNA viral load in infections with the Delta variant. On the other hand, lower viral loads were reported in patients infected with Omicron BA.1 than in those infected with Delta. Infections with Omicron BA.2 lead to higher levels of RNA viral loads than with BA.1 (reviewed in^22^). These observations are consistent with the trends in the median Ct values at the end of 2021, as the 3rd wave was preceded by stably increasing median Ct values and thus would not be predicted. Starting from 2022-06, the median Ct values gradually decreased with the emergence of BA.2 variant and concomitant changes in the state policy reducing population testing and thus possibly increasing the proportion of tests in symptomatic individuals. Although comparison of viral loads between symptomatic and asymptomatic individuals is challenging, some studies showed higher viral loads in symptomatic individuals^23–26^.

Second, we analysed the distribution of Ct values in different age groups with an aim to identify groups that might contribute most to the spread of infection. Several studies have addressed the effect of age on viral loads and the results are highly contradicting^27^. In aggregate, our data revealed that children consistently had a 4 to 5-fold reduction in viral RNA load (as estimated by Ct) and two-times lower test positivity rate than adults. This corresponds with the findings that children are less susceptible to SARS-CoV-2 infection^11,28^, tend to be asymptomatic or paucisymptomatic compared to adults^15,26^ and are rarely the index case in household transmission chains^29,30^. Children also have a lower risk of reinfection than adults^31^. Comparison with adults could be potentially complicated by differential exposure risks over time, but the analysis of odds ratios and differences in Ct values week-by-week did not indicate that the aggregate values were a result of prolonged periods of school closures. Although the Ct values and odds ratios oscillated, the trends remained stable. Despite all these findings, SARS-CoV-2 testing in the Czech Republic was aimed disproportionately at children. It should be noted that higher Ct values in children could be caused by different viral kinetics (e. g. lower peak viral load and/or faster clearance) but also by differences in the epidemic trajectories. Our data are not suitable to assess the involvement of these factors.

Third, we assessed the outcome of saliva tests that have been performed as part of a mandatory surveillance testing program at schools. The rarity of positive results indicates that this testing was not cost-effective and did not prevent spread of SARS-CoV-2. Our results were consistent with another investigation showing that children and teachers do not contribute significantly to the spread of the disease via attendance in educational settings when epidemic control strategies and effective testing for the population exist^32^. A study of the COVID-19 epidemics indicated that the adults aged 20–49 are the major contributors to the spread of the disease, before and after school reopening^5^.

In general, this investigation demonstrates several ways in which Ct values could be helpful in the study of epidemics. First, early measurement of Ct values can identify which subgroups are more likely to be affected by the virus. The viral load of SARS-CoV-2 in the upper respiratory tract is considered to be a proxy for transmission risk (reviewed in^22^). In our population, early studies would have indicated the low viral load in children, equivalent results when stratified by sex, and higher viral load in older individuals. Second, falling median Ct values can indicate an upcoming epidemic growth but increasing median Ct values do not exclude it. Health system authorities should consider collecting weekly median Ct values of positively tested samples from major diagnostic laboratories for regional epidemic surveillance. Also, results of methods that estimate the epidemic dynamics based on Ct values can be improved if they incorporate information about the age of positive individuals. Further studies should be performed to determine if Ct values varied by other important COVID-19 risk factors, including obesity, race, cancer, and immunosuppression.

## Supplementary Information

Supplementary Information 1.

Supplementary Figures.

Supplementary Tables.

## Data Availability

Our data are accessible to researchers upon reasonable request to the corresponding author.

## References

[1] Bullard J (2021). Infectivity of severe acute respiratory syndrome coronavirus 2 in children compared with adults. CMAJ.

[2] Lyngse FP (2021). Association between SARS-CoV-2 transmission risk, viral load, and age: A Nationwide Study in Danish Households. medRxiv.

[3] Tom MR, Mina MJ (2020). To interpret the SARS-CoV-2 test, consider the cycle threshold value. Clin. Infect. Dis..

[4] Liu Y (2020). Viral dynamics in mild and severe cases of COVID-19. Lancet Infect. Dis..

[5] Monod M (2021). Age groups that sustain resurging COVID-19 epidemics in the United States. Science.

[6] AACC. *AACC Recommendation for Reporting SARS-CoV-2 Cycle Threshold (CT) Values*, https://www.aacc.org/science-and-research/covid-19-resources/statements-on-covid-19-testing/aacc-recommendation-for-reporting-sars-cov-2-cycle-threshold-ct-values (2021).

[7] Hay JA (2021). Estimating epidemiologic dynamics from cross-sectional viral load distributions. Science.

[8] Walker AS (2021). Ct threshold values, a proxy for viral load in community SARS-CoV-2 cases, demonstrate wide variation across populations and over time. Elife.

[9] Yin N (2021). Leveraging of SARS-CoV-2 PCR cycle thresholds values to forecast COVID-19 trends. Front. Med.-Lausanne.

[10] Lin Y (2022). Incorporating temporal distribution of population-level viral load enables real-time estimation of COVID-19 transmission. Nature Commun..

[11] Viner RM (2021). Susceptibility to SARS-CoV-2 infection among children and adolescents compared with adults a systematic review and meta-analysis. JAMA Pediatr..

[12] Irfan O, Li J, Tang K, Wang Z, Bhutta ZA (2021). Risk of infection and transmission of SARS-CoV-2 among children and adolescents in households, communities and educational settings: A systematic review and meta-analysis. J. Glob. Health.

[13] Bertozzi AL, Franco E, Mohler G, Short MB, Sledge D (2020). The challenges of modeling and forecasting the spread of COVID-19. Proc. Natl. Acad. Sci. USA.

[14] Lipsitch M, Swerdlow DL, Finelli L (2020). Defining the epidemiology of Covid-19—studies needed. N. Engl. J. Med..

[15] Davies NG (2020). Age-dependent effects in the transmission and control of COVID-19 epidemics. Nat. Med..

[16] Trang NV (2015). Determination of cut-off cycle threshold values in routine RT-PCR assays to assist differential diagnosis of norovirus in children hospitalized for acute gastroenteritis. Epidemiol. Infect..

[17] Ochoa V (2022). Infants younger than 6 months infected with SARS-CoV-2 show the highest respiratory viral loads. J. Infect. Dis..

[18] Yang D (2022). Bimodal distribution pattern associated with the PCR cycle threshold (Ct) and implications in COVID-19 infections. Res. Square..

[19] Piubelli C (2021). Overall decrease in SARS-CoV-2 viral load and reduction in clinical burden: The experience of a hospital in northern Italy. Clin. Microbiol. Infect..

[20] Jones TC (2021). Estimating infectiousness throughout SARS-CoV-2 infection course. Science.

[21] Bushman M, Kahn R, Taylor BP, Lipsitch M, Hanage WP (2021). Population impact of SARS-CoV-2 variants with enhanced transmissibility and/or partial immune escape. Cell.

[22] Puhach O, Meyer B, Eckerle I (2022). SARS-CoV-2 viral load and shedding kinetics. Nat. Rev. Microbiol.

[23] Kociolek LK (2021). Comparison of upper respiratory viral load distributions in asymptomatic and symptomatic children diagnosed with SARS-CoV-2 infection in pediatric hospital testing programs. J. Clin. Microbiol..

[24] Zhou R (2020). Viral dynamics in asymptomatic patients with COVID-19. Int. J. Infect. Dis..

[25] Hall SM (2022). Comparison of anterior nares CT values in asymptomatic and symptomatic individuals diagnosed with SARS-CoV-2 in a university screening program. PLoS ONE.

[26] Chung E (2021). Comparison of symptoms and RNA levels in children and adults with SARS-CoV-2 infection in the community setting. JAMA Pediatr..

[27] Walsh KA (2020). SARS-CoV-2 detection, viral load and infectivity over the course of an infection. J. Infect..

[28] Gudbjartsson DF (2020). Spread of SARS-CoV-2 in the icelandic population. N. Engl. J. Med..

[29] Zhu YS (2021). A meta-analysis on the role of children in severe acute respiratory syndrome coronavirus 2 in household transmission clusters. Clin. Infect. Dis..

[30] Maltezou HC (2020). Children and adolescents with SARS-CoV-2 infection epidemiology, clinical course and viral loads. Pediatr. Infect. Dis. J..

[31] Mensah AA (2022). Risk of SARS-CoV-2 reinfections in children: A prospective national surveillance study between January, 2020, and July, 2021, in England. Lancet Child Adolesc. Health.

[32] Macartney K (2020). Transmission of SARS-CoV-2 in Australian educational settings: A prospective cohort study. Lancet Child Adolesc. Health.

