# Supplementary material for: Trends in SARS-CoV-2 cycle threshold values in the Czech Republic from April 2020 to April 2022
Source: Sci Rep. 2023 Apr 15;13:6156. doi: 10.1038/s41598-023-32953-2 (PMC10105352; doi:10.1038/s41598-023-32953-2)
Supplement: Supplementary file 1 — Supplementary Information 1. [file 41598_2023_32953_MOESM1_ESM.docx]

**Supplementary Appendix**

This appendix has been provided by the authors to give readers additional information about their work.

Supplement to: Musalkova D, Piherova L, Kwasny O, et al.: Trends in SARS-CoV-2 Cycle Threshold Values in the Czech Republic from April 2020 to April 2022

**Supplementary methods**

The viral nucleic acids were extracted by viRNAtrap extraction kit (GeneSpector, Czech Republic) using KingFisher Flex Purification System (Thermo Scientific, Waltham, MA, USA). 200 µL of a sample from nasopharyngeal swab in viRNAtrap medium or saliva from Salivette tube (Sarstedt, Nümbrecht, Germany) was used for the extraction process. RT-qPCR was performed using “gb SARS-CoV-2 Combi” kit (Generi Biotech, Czech Republic) and an exogenous internal control was added to each sample prior to extraction, as recommended by the detection kit manufacturer. 5 µL of RNA eluate was used for each reaction (total elution volume was 50 µL) and all sample were tested in singlets. Both genes (RdRP and E) were labelled by the same fluorophore for detection in one channel (FAM) in one reaction. Data were acquired with CFX96 System (Bio-Rad, Hercules, CA, USA) and analysed using CFX Manager 3.1 software (Bio-Rad, Hercules, CA, USA). The presence of SARS-CoV-2 was determined by the value of the cycle threshold Ct in FAM channel and the internal control, present throughout the complete process of isolation, in HEX channel. A positive result, indicating the presence of SARS-CoV-2 RNA, was determined as cycle threshold (Ct) values less than 38 as recommended by the manufacturer. If the internal control has the cycle threshold (Ct) value lower than 35 and the FAM channel detecting the presence of SARS-CoV-2 has a negative signal, the result was negative with either no presence of the virus in the sample or a concentration below the level of detection. If both channels had no signal, the reaction and the result was invalid.

On each tested plate three types of controls were included; negative and positive controls as was recommended by detection kit manufacturer and internal laboratory controls (two negative samples) that were randomly inserted on plate before extraction.

No substantial changes were made in the laboratory protocols, or in the sample collection over the analysed time period.
