# Supplementary material for: Trends in SARS-CoV-2 cycle threshold values in the Czech Republic from April 2020 to April 2022
Source: Sci Rep. 2023 Apr 15;13:6156. doi: 10.1038/s41598-023-32953-2 (PMC10105352; doi:10.1038/s41598-023-32953-2)
Supplement: Supplementary file 2 — Supplementary Figures. [file 41598_2023_32953_MOESM2_ESM.pdf]

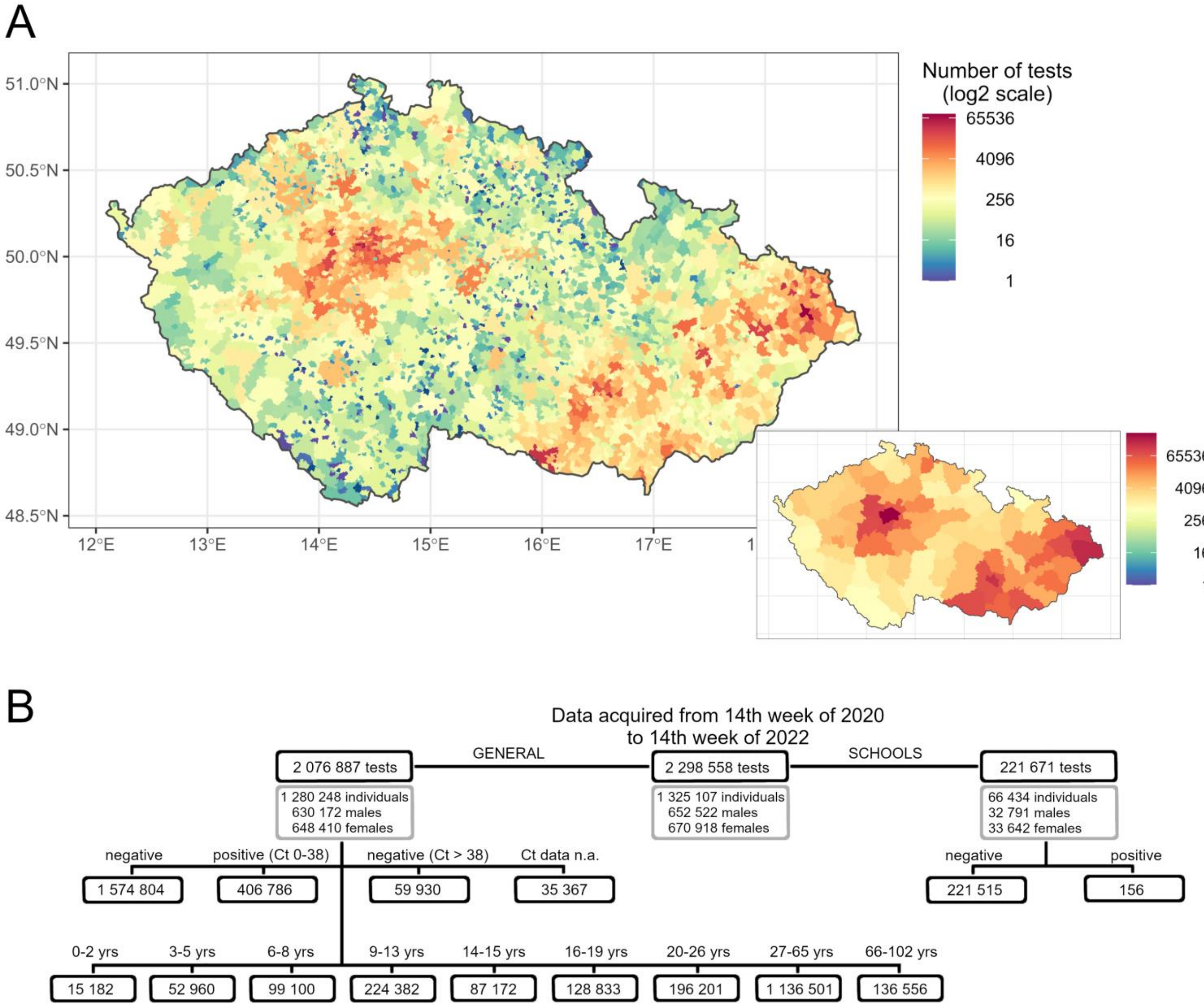

Supplementary Figure 1

(A) The population source for the study. Postal codes and the corresponding number of performed tests (all tests combined) were mapped to the Czech Republic using R package RCzechia (version 1.9.1, https://github.com/jlacko/RCzechia). Zip codes with 0 performed tests are shown in dark blue. The inset shows the aggregated data for all 76 districts of the Czech Republic. **(B)** The workflow diagram and summary of the data. The numbers in black frames show the number of performed tests in the corresponding groups. The samples for general testing (nasopharyngeal swabs) were collected viRNAtrap medium. The samples for school testing (saliva) were collected in Salivette tubes. Data from surveillance school testing performed in the saliva samples is shown separately, in the Figure 4 only.

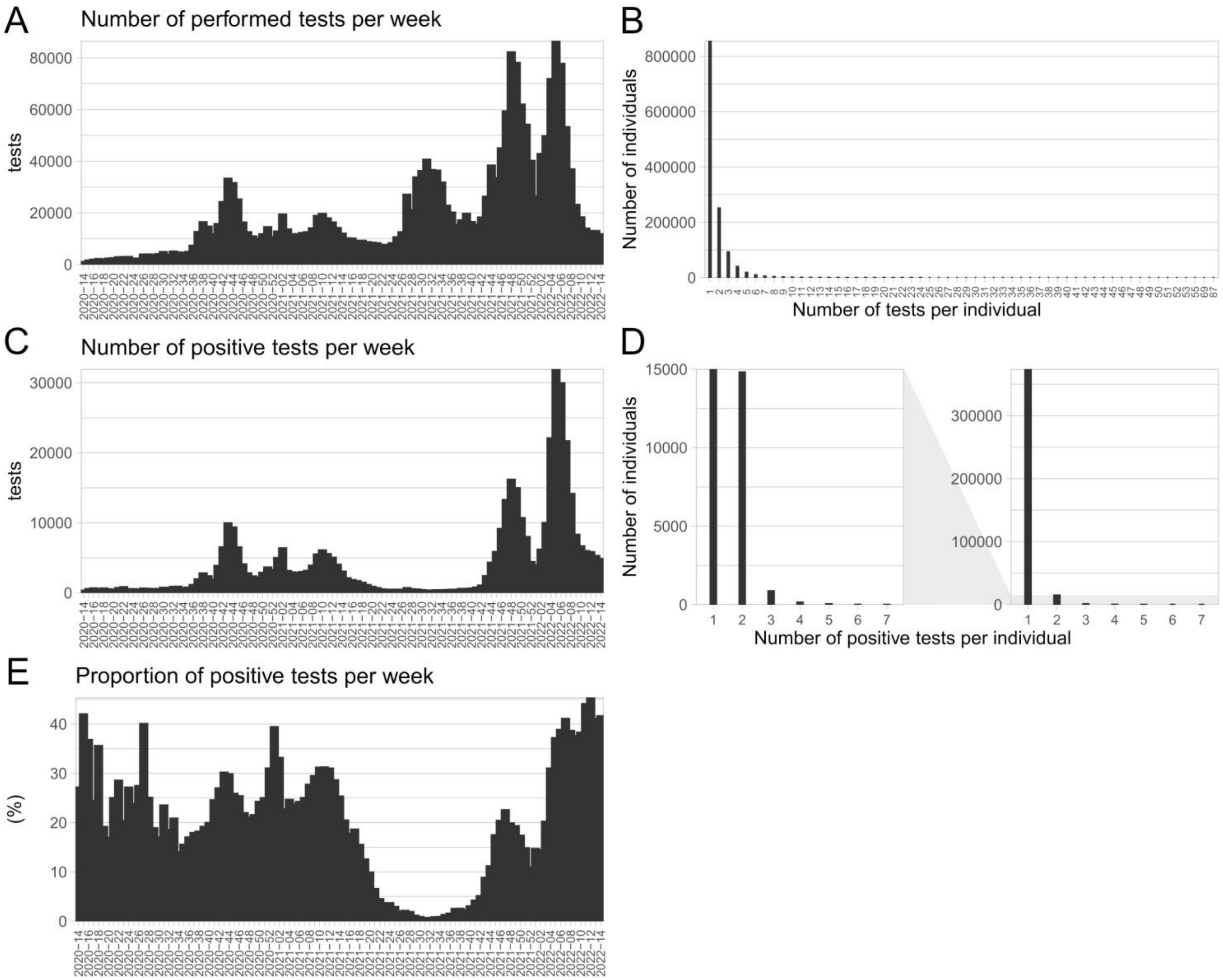

Supplementary Figure S2

The distribution of all tests (nasopharyngeal swabs) performed per week **(A)** and the corresponding number of all positive tests **(C)**, together with distributions of all performed tests **(B)** and positive tests **(D)** per individual. **(E)** Proportion of positive tests per week.

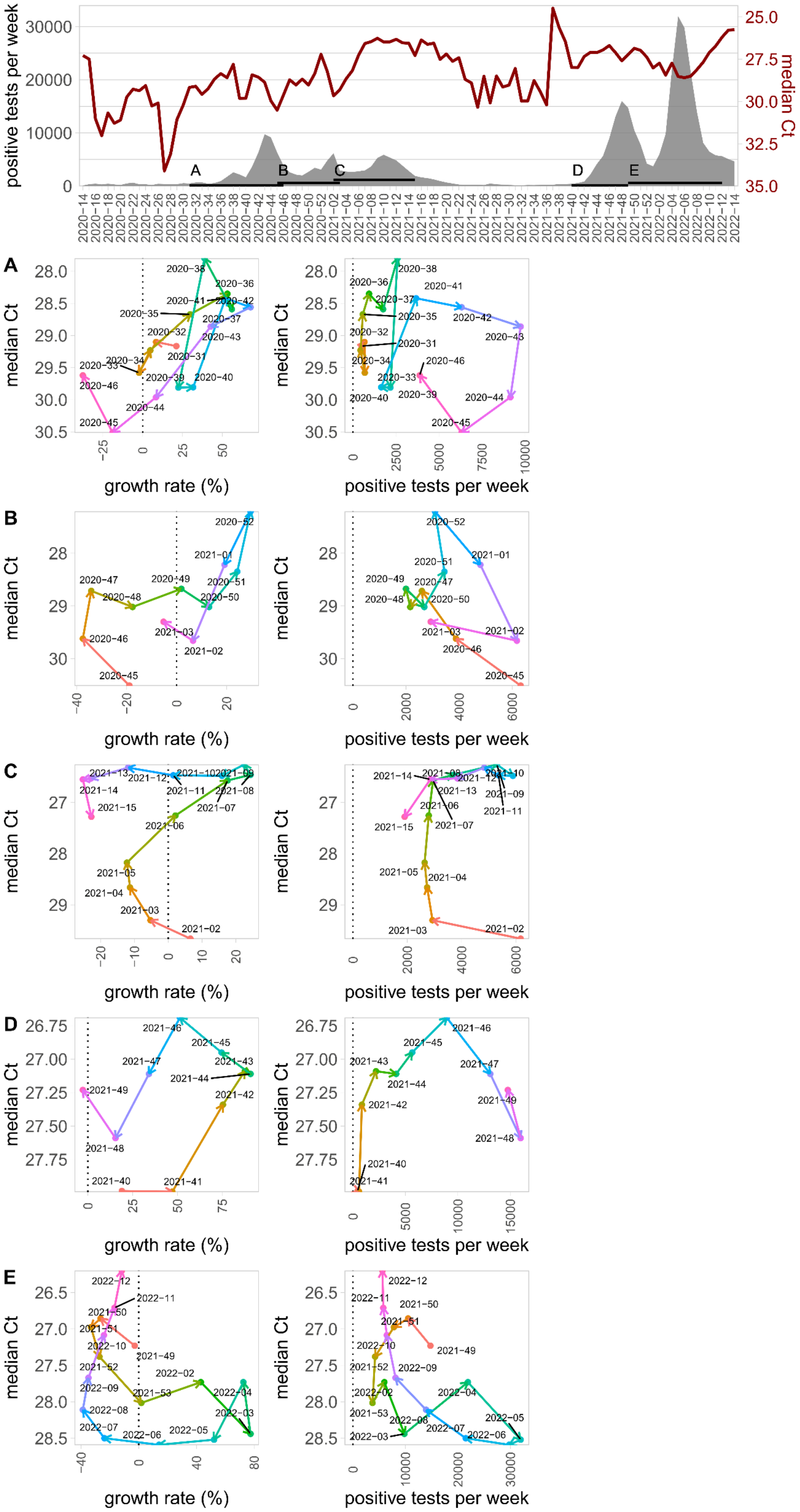

Supplementary Figure S3

(A,B,C,D, and E) Connected scatterplots showing the correlation between median Ct and growth rate (smoothed values as shown in the Figure 1C) and between median Ct and number of positive tests per week. The points are connected with a line in temporal sequence. The parts A, B, C, D, and E correspond to the time periods highlighted in the graph in the upper part.

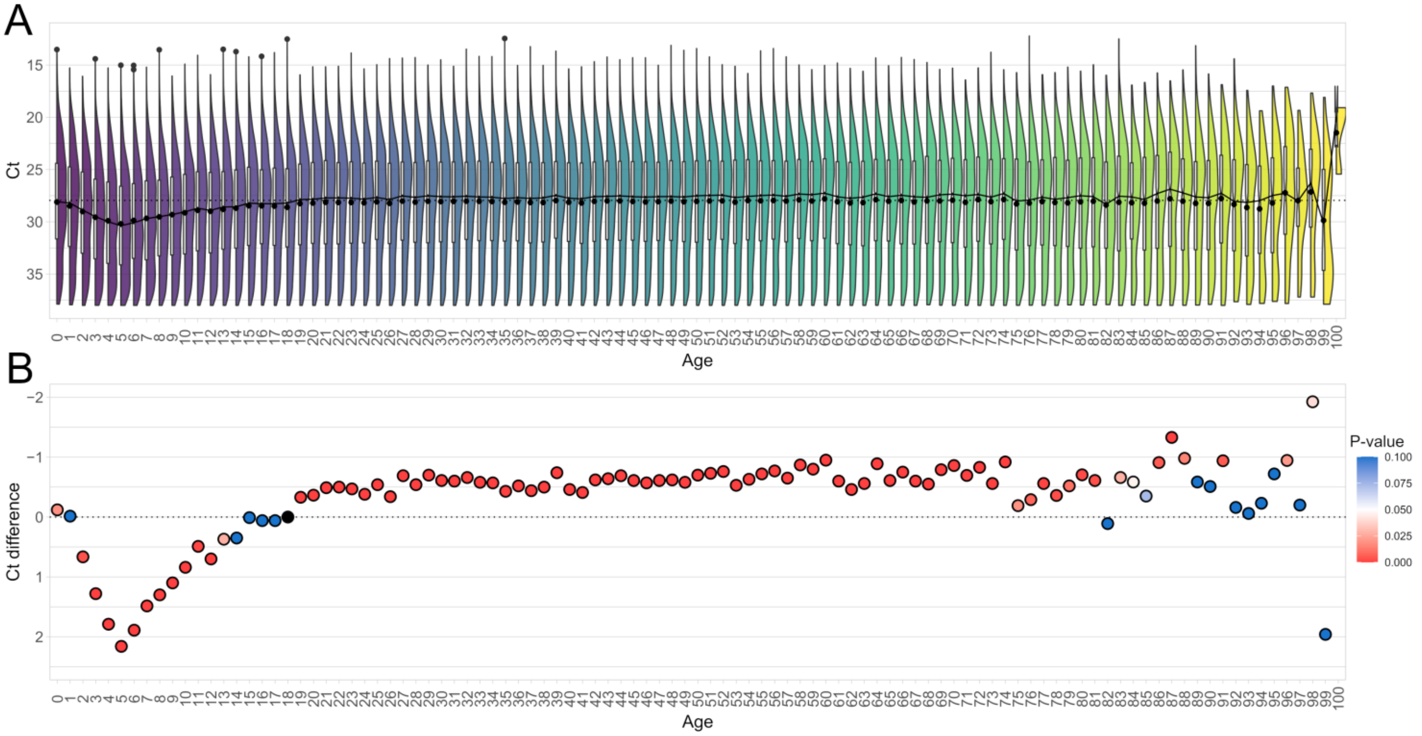

Supplementary Figure S4
(A) The distribution of Ct values of positive tests categorized acccording to the age of the individual. **(B)** Differences of median Ct values (compared to the age of 18 yrs). Color of the points corresponds to P-values from Dunn´s test.

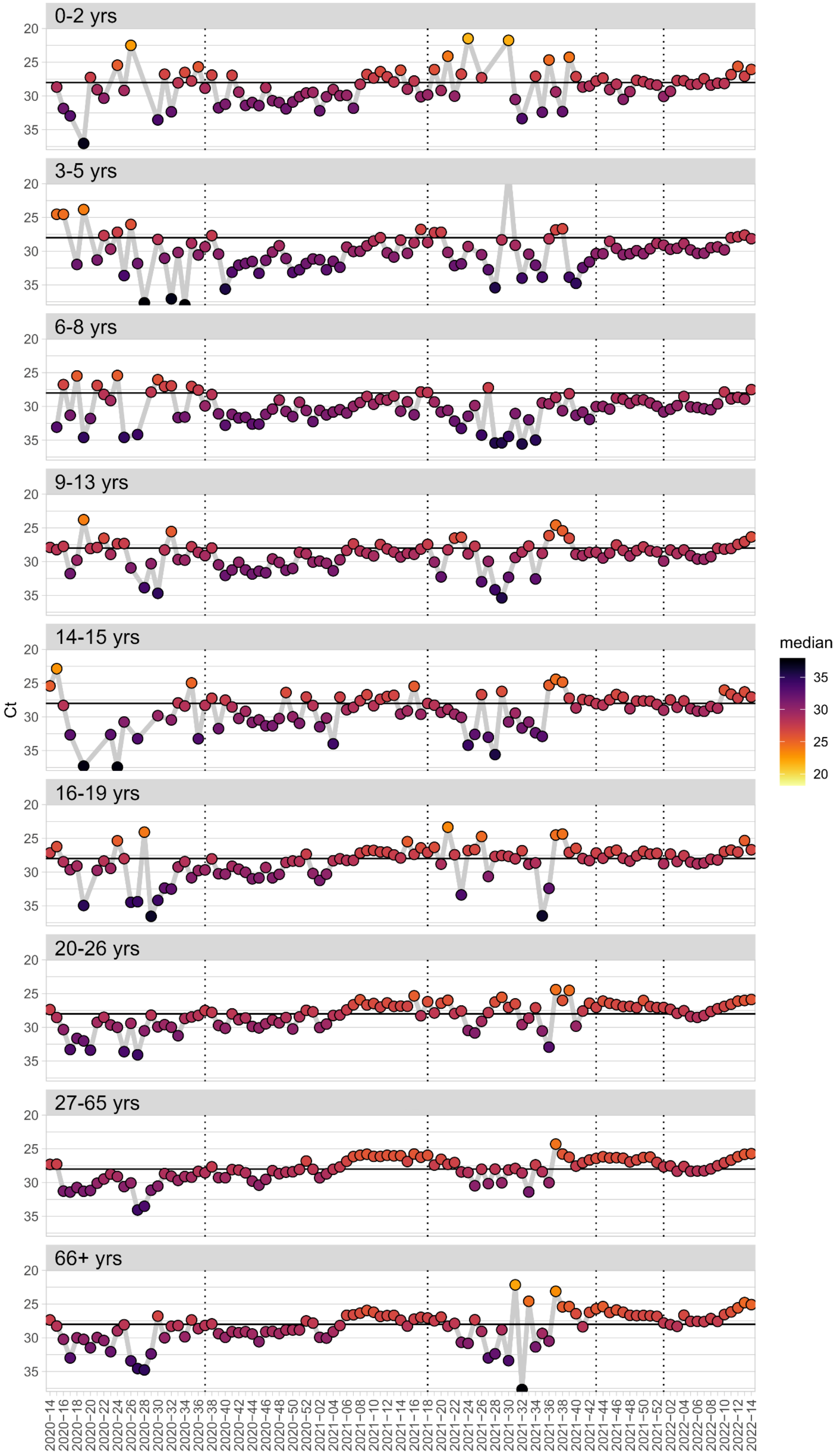

Supplementary Figure S5

Weekly median Ct values in different age groups. The colour of the points corresponds to the median Ct value. Dotted vertical lines delimit the weeks 2020-37, 2021-18, 2021-43, and 2022-01 and correspond to the timing of the waves as defined in the Figure 1.

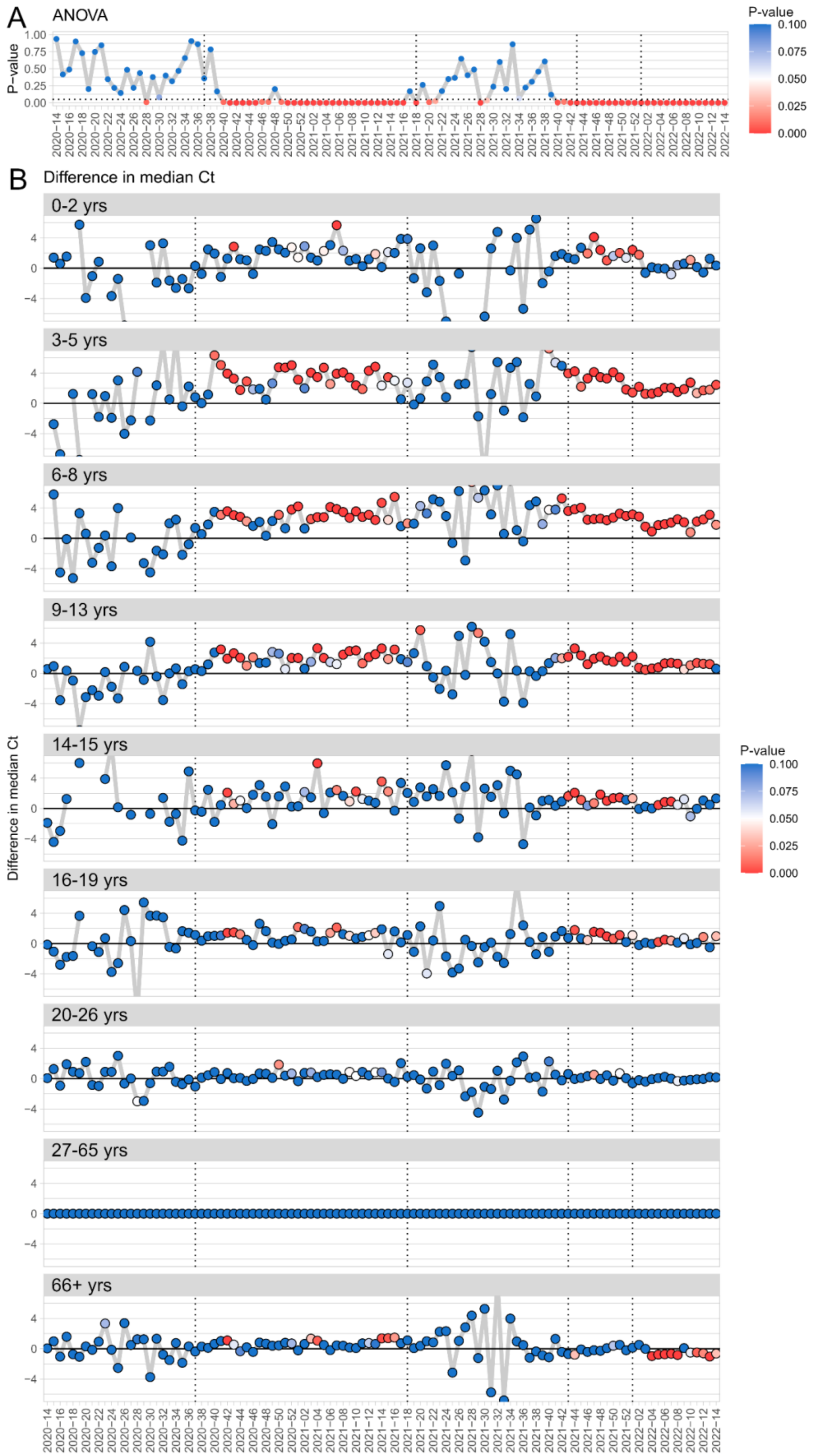

Supplementary Figure S6

Weekly differences in median Ct values. **(A)** P-values from Kruskal-Wallis One-Way Analysis of Variance performed weekly. **(B)** Differences in median Ct values of positive tests for every week. Group of adults (27- 65 yrs) is the reference. The colour of the points corresponds to the P-value from Dunn's post hoc test. Dotted vertical lines mark weeks 2020-37, 2021-18, 2021-43, and 2022-01 and correspond to the timing of the waves as defined in the Figure 1. The number of positive tests was over 1000 per week in the time period between weeks 2020-37 and 2021-18, and between 2021-43 and 2022-14.

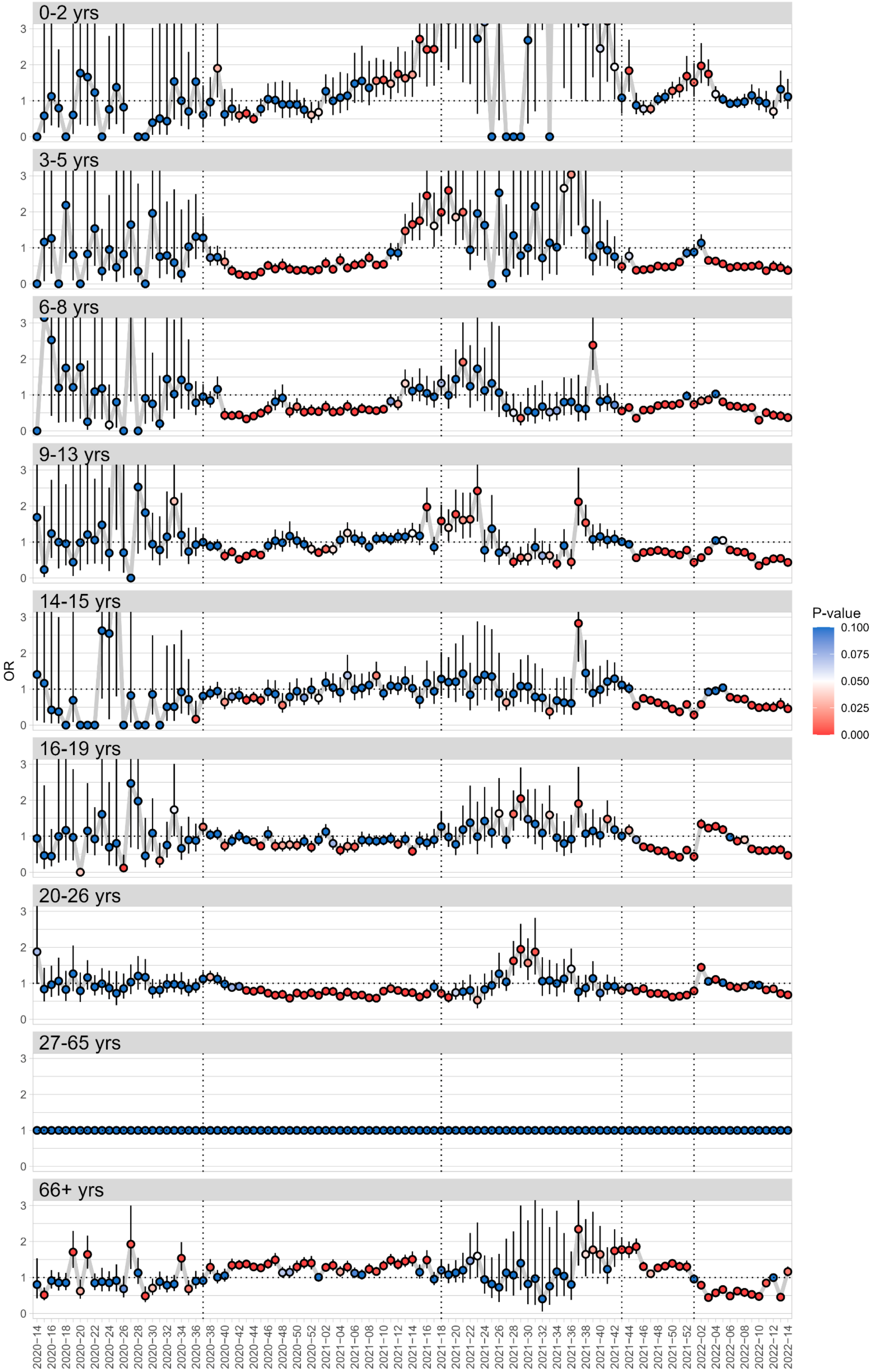

Supplementary Figure S7

Odds ratios of diagnostic test positivity rate per week and age group compared to the group of adults (27-65 yrs). Confidence intervals were computed using the Wald method, P-values correspond to the Fisher´s exact test. Dotted vertical lines delimit the weeks 2020-37, 2021-18, 2021-43, and 2022-01 and correspond to the timing of the waves as defined in the Figure 1.

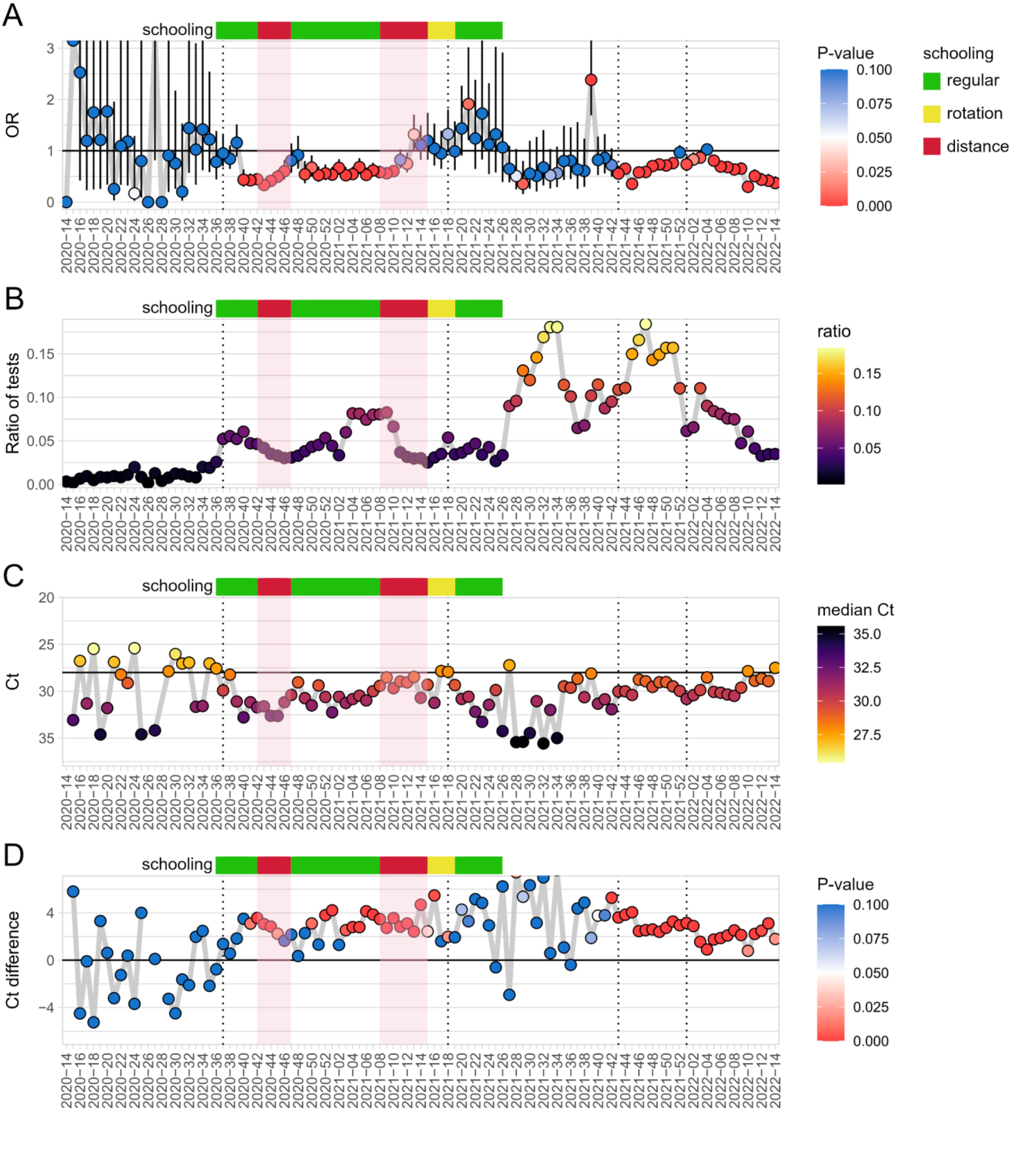

Supplementary Figure S8

Summary for the group of 6-8 yrs old. **(A)** Odds ratios of diagnostic test positivity rate compared to the group of adults (27-65 yrs). Confidence intervals were computed using the Wald method, P-values correspond to the Fisher´s exact test. **(B)** The ratio of total tests performed in the group of 6-8 yrs old vs. 27-65 yrs old. **(C)** Weekly median Ct values. The colour of the points corresponds to the median Ct value. **(D)** Differences of median Ct values of positive tests for every week. The colour of the points corresponds to the P-value from Dunn's post hoc test. Group of adults (27- 65 yrs) is the reference.

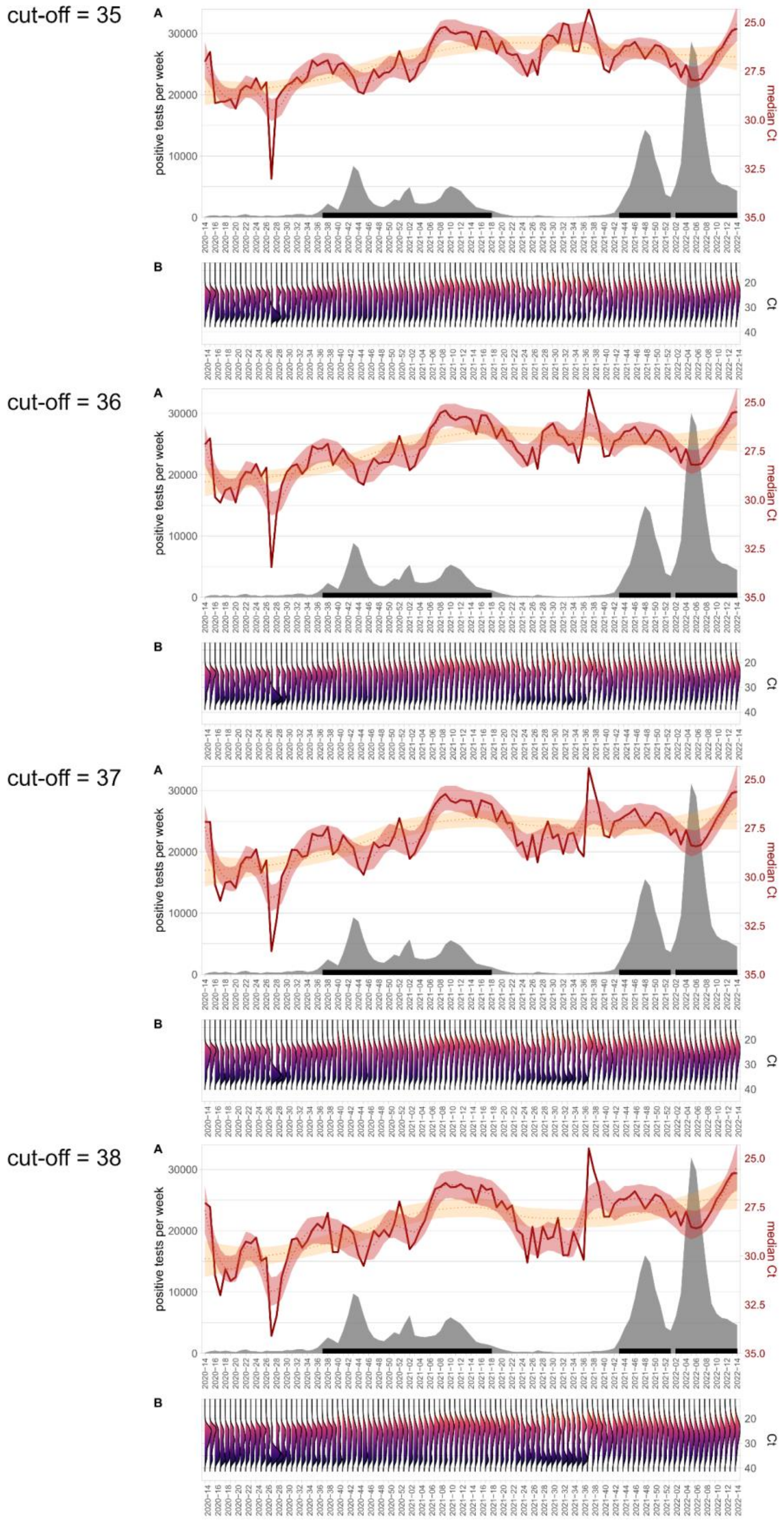

Supplementary Figure S9

Four versions of Figure 1 produced with different cut-off values for the test positivity. **(A)** The time course of median Ct values per week (dark red line) and the total number of positive tests per week (grey area). Loess curves fitted to Ct medians are shown as a dotted red line (alpha=0.15) and yellow line (alpha=0.7). **(B)** Ct distributions of positive tests shown as half violin plots.

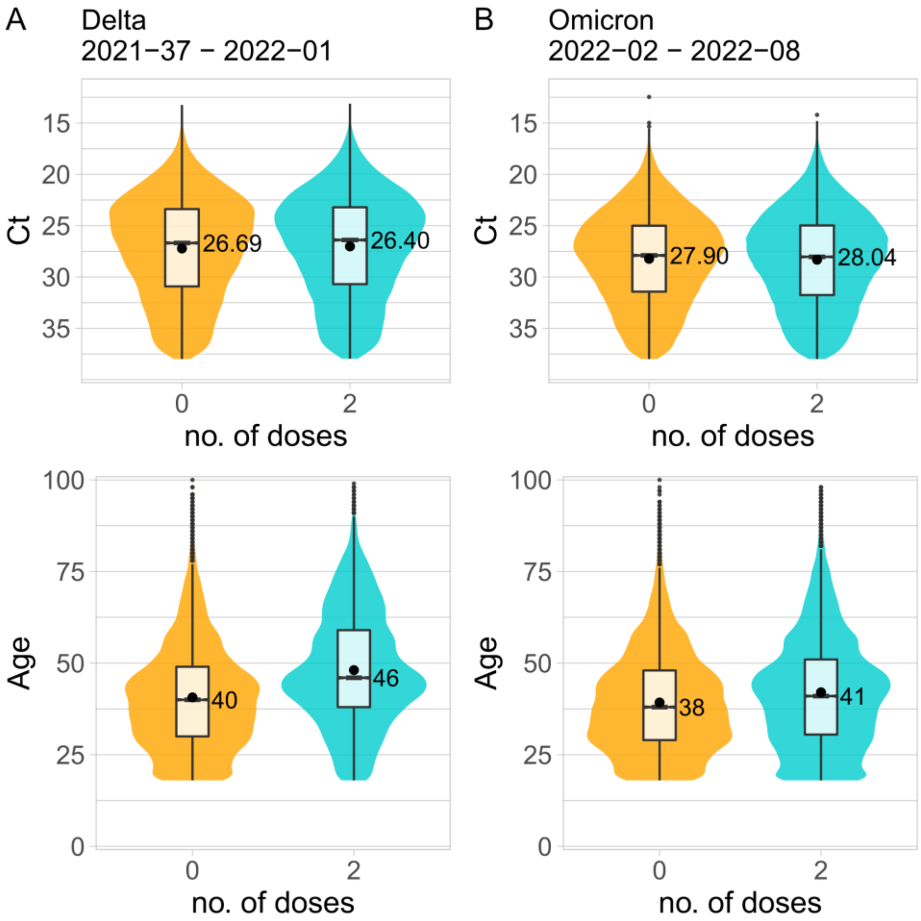

Supplementary Figure S10

Distributions of Ct values of positive samples and the age of the individuals in the period between 2021-37 and 2022-01 corresponding to the Delta wave **(A)** and the period between 2022-02 and 2022-08 corresponding to the Omicron wave **(B)**. Only the results of adult individuals that were either unvaccinated or fully vaccinated were included in the comparison. Median Ct value and median age is shown alongside the corresponding box plot.
